# Preservation of vascular endothelial repair in mice with diet‐induced obesity

**DOI:** 10.1002/osp4.282

**Published:** 2018-06-26

**Authors:** S. T. Rashid, N. J. Haywood, N. Y. Yuldasheva, J. Smith, A. Aziz, D. J. A. Scott, M. T. Kearney, S. B. Wheatcroft

**Affiliations:** ^1^ Leeds Institute of Cardiovascular and Metabolic Medicine, Faculty of Medicine and Health University of Leeds Leeds UK; ^2^ Department of Vascular Surgery Manchester University NHS Foundation Trust Manchester UK; ^3^ Division of Diabetes, Endocrinology and Gastroenterology University of Manchester Manchester UK; ^4^ Manchester Academic Health Science Centre Manchester UK; ^5^ Leeds Vascular Institute Leeds Teaching Hospitals NHS Trust Leeds UK

**Keywords:** Diet‐induced obesity, endothelial regeneration, glucose intolerance, insulin resistance

## Abstract

**Introduction:**

Preservation of structural integrity of the endothelial monolayer and maintenance of endothelial cell function are of critical importance in preventing arterial thrombosis, restenosis and atherosclerosis. Obesity has been intimately linked with endothelial dysfunction, and reports of reduced abundance and functional impairment of circulating progenitor cells in obesity have led to the suggestion that defective endothelial repair contributes to obesity‐related cardiovascular disease.

**Methods:**

C57BL/6 mice were fed a high‐fat diet for either 3 or 6 months to induce obesity; metabolic phenotyping was then carried out before femoral artery wire injury was performed. Endothelial regeneration was then quantified. Mononuclear cells and myeloid angiogenic cells were cultured and characterized for pro‐angiogenic properties.

**Results:**

No impairment of endothelial regeneration following mechanical endothelial injury in diet‐induced obese mice when compared with chow‐fed controls was observed, despite the induction of an adverse metabolic phenotype characterized by glucose intolerance and insulin resistance. Dietary‐obese mice had increased numbers of circulating myeloid angiogenic cells, which retained normal functional properties including intact paracrine angiogenic effects.

**Conclusion:**

Preserved endothelial regeneration despite metabolic dysregulation in dietary obese mice suggests that compensatory mechanisms mitigate the deleterious influence of insulin resistance on endothelial repair in obesity.

## Introduction

Recent changes in nutrition and lifestyle have provoked a significant increase in the prevalence of obesity, which is associated with reduced longevity and increased lifetime risk of morbidity and mortality from cardiovascular disease 1. Functional and structural integrity of the endothelial monolayer plays a critical role in vascular homeostasis. Regeneration of damaged endothelium following injury is essential to prevent adverse remodelling 2 and is mediated by two broad mechanisms: proliferation and migration of local endothelial cells 3 and recruitment of circulating cells to the injured vessel 4. We previously showed that insulin resistance in lean mice significantly impaired endothelial regeneration following denuding injury 5. However, the effects of obesity on endothelial regeneration have not previously been studied. Here, endothelial repair following femoral artery wire injury in mice subjected to diet‐induced obesity was examined.

Preservation of structural integrity of the endothelial monolayer and maintenance of endothelial cell function are of critical importance in preventing arterial thrombosis, restenosis and atherosclerosis. Obesity has been intimately linked with endothelial dysfunction, not least through its association with insulin resistance 6. In health, biochemical or physical endothelial injury is offset by reparative processes orchestrated by proliferation of vessel wall endothelial cells 3 and the recruitment of pro‐reparative circulating cells 4. The latter have recently been redefined to include myeloid angiogenic cells (MACs), which are of haematopoetic lineage and promote repair by secretion of paracrine angiogenic factors, and endothelial colony forming cells, which are of true endothelial lineage and participate directly in vascular endothelial repair 7. A large body of evidence from pre‐clinical and clinical studies indicates that cells implicated in endothelial repair are less abundant and are dysfunctional in the context of obesity 8, 9, 10, 11. The consequent anticipated defects in endothelial regenerative capacity have been proposed as an important contributor to the increased risk of cardiovascular disease in obesity 11.

## Materials and methods

### Animal husbandry

Male C57BL/6J mice were purchased from Charles River Laboratories, Cambridge, UK. Experiments were carried out under the authority of UK Home Office Licence PPL40/3523. Mice were group housed in cages of five animals. Cages were maintained in humidity‐controlled and temperature‐controlled conditions (humidity 55% at 22 °C) with a 12‐h light–dark cycle. To induce obesity, mice received free access to a high‐fat diet (60% of energy from fat) (D12492, Research Diets, New Brunswick, NJ, USA) with the following composition: protein 26.2%, fat 34.9% and carbohydrate 34.9%. Control animals received standard chow diet (BK001; Special Diet Services, Essex, UK) with the following composition: protein 19.64%, fat 7.52%, ash 6.21%, moisture 10%, fibre 3.49%, nitrogen‐free extract 54.90% and energy 3.29 kcal g^−1^.

Mice were established on experimental or control diets from 8 weeks of age. Metabolic phenotyping, blood sampling and arterial injury experiments were performed at ~3 months between 12 and 14 weeks of feeding (at 20–24 weeks old). A separate group of mice underwent metabolic phenotyping and arterial injury experiments at ~6 months between 26 and 28 weeks of feeding (at 34–36 weeks old).

### Metabolic profiling

Mice were fasted for 16 h prior to glucose tolerance test or 2 h prior to insulin tolerance test. Blood glucose was measured using a hand‐held glucose meter (Accu‐Chek Aviva, Roche Diabetes Care UK, Burgess Hill, West Sussex, UK) at intervals following ip administration of glucose (1 mg g^−1^) or recombinant human insulin (Actrapid; Novo Nordisk, Bagsvaerd, Denmark; 0.75 IU kg^−1^). Mice were not restrained between measurements.

To obtain plasma for insulin levels, blood was collected from the lateral saphenous vein (EDTA collection tubes Sarstedt 16.444, Nümbrecht, Germany). Samples were then spun at 10,000 rpm for 10 min in a benchtop centrifuge. Plasma was stored at −20 °C until use in an ultra‐sensitive mouse insulin ELISA (90080, Crystal Chem, Downers Grove, IL, USA) as per kit instructions.

### Arterial injury

Mice were anaesthetized with isoflurane (2.5–5%) before a small incision was made in the mid‐thigh to permit isolation of the femoral artery. Following an arteriotomy made using iris scissors (World‐Precision Instruments, Sarasota, FL, USA), a 0.014‐in.‐diameter angioplasty guide wire with a tapered tip (Hi‐torque Cross‐it XT; Abbott‐Vascular, Abbott, IL, USA) was introduced. The angioplasty guide wire was advanced 2 cm, and three passages were performed per mouse, resulting in complete arterial denudation. The guide wire was removed, and the suture was tightened rapidly. The vessel was then ligated, and the skin was closed with a continuous suture. The contralateral artery underwent an identical sham operation, without passage of the wire. Animals received post‐operative analgesia with buprenorphine (0.25 mg kg^−1^). Mice were anesthetized 7 d after wire injury, and 50 μL of 0.5% Evans blue dye was injected into the inferior vena cava. The mice were perfused/fixed with 4% paraformaldehyde in phosphate buffered saline (PBS) before the femoral arteries (injured and uninjured) were harvested. The vessels were opened longitudinally. The areas stained and unstained in blue were measured in a 5‐mm injured segment beginning 5 mm distal to the aortic bifurcation, and the percentage areas were calculated using imageproplus7.0 software (Media Cybernetics, Bethesda, MD, USA) as previously described 5, 11, 12.

## Cell culture

Mononuclear cells (MNCs) were isolated from 1 mL of blood, obtained from the vena cava under terminal anaesthesia by density gradient centrifugation (Histopaque‐1083; Sigma‐Aldrich Company Ltd, Gillingham, Dorset, UK). MNCs were seeded on fibronectin 24‐well plates (BD Biosciences, Wokingham, Berkshire, UK) at a density of 1 × 10^6^ cells per well and cultured in endothelial cell growth (EGM‐2) medium supplemented with EGM‐2 Bullet kit (Lonza, Basel, Switzerland) in addition to 20% foetal calf serum.

Spleens obtained from mice under terminal anaesthesia were minced mechanically. MNCs were isolated by density gradient centrifugation, as described earlier. After washing steps, cells were seeded on fibronectin 24‐well plates at a seeding density of 8 × 10^6^ cells per well and cultured as described earlier.

Tibias and femurs were flushed three times in DMEM with a 26‐gauge needle to collect bone marrow. MNCs were isolated by density gradient centrifugation as described earlier. After washing steps, cells were seeded on fibronectin 24‐well plates at a seeding density of 1 × 10^6^ cells per well and cultured as described earlier.

After 7‐d incubation at 37 °C in 5% CO_2_, non‐adherent cells were discarded by gentle washing with PBS and adherent cells were re‐suspended in medium. At day 5, conditioned medium from bone marrow‐derived MNCs was taken for tube‐forming assays. At day 7, attached cells from peripheral blood, spleen, and bone marrow were stained for the uptake of 1,1′‐dioctadecyl‐3,3,3′,3′‐tetramethylindocarbocyanine‐labelled acetylated low‐density lipoprotein (DiI‐Ac‐LDL) (Molecular Probes, Invitrogen, Carlsbad, CA, USA) and lectin from Ulex europaeus fluorescein isothiocyanate conjugate (Sigma). Cells were first incubated with DiI‐Ac‐LDL at 37 °C for 3 h and later fixed with 4% paraformaldehyde for 10 min. Cells were washed and reacted with lectin for 1 h. After the staining, cells were quantified by examining 10 random high‐power fields (HPFs), and double‐positive cells were identified as MACs.

### Tube forming

The potential for MNCs to stimulate angiogenesis by secreting paracrine factors was assessed in conditioned media obtained as described earlier. Human umbilical vein endothelial cells (Promocell, C‐12203) were seeded at 100,000 cells per well of a matrigel‐coated (Becton Dickinson, 734‐0268) 24‐well plate and incubated for 24 h at 37 °C in conditioned media. Endothelial tube formation was evaluated as the mean number of tubes formed per HPF as previously described 5, 11, 12.

### Adhesion

Fifty thousand spleen‐derived MACs were re‐suspended in EGM‐2 medium, plated onto 24‐well plates coated with fibronectin and incubated for 1 h at 37 °C. After being washed three times with PBS, attached cells were counted. Adhesion was evaluated as the mean number of attached cells per HPF (×100).

### Blood pressure

Blood pressure was measured in conscious animals by tail‐cuff system (CODA4, Kent Scientific Corporation, Torrington, CT, USA).

### Data analysis

Data are shown as mean ± standard error of the mean. Statistical analysis was performed using graphpad prism (GraphPad Software, La Jolla, CA, USA) and Student's unpaired *t*‐test.

## Results

### Diet‐induced obesity results in glucose intolerance and insulin resistance

**Figure 1:**
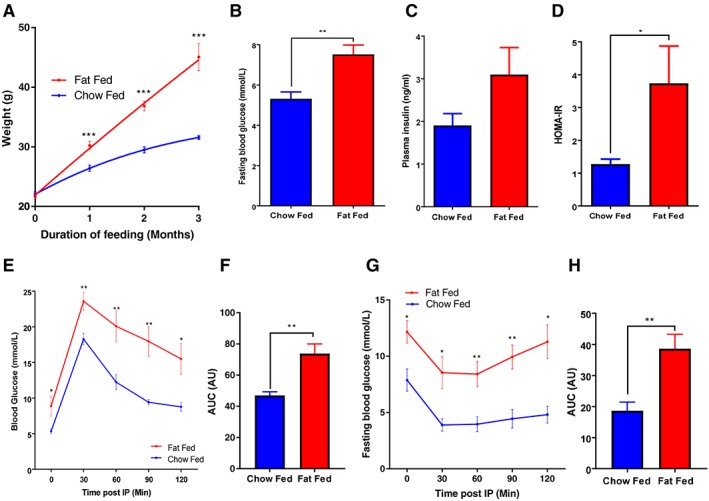
Body mass increased significantly more in diet‐induced obese mice compared with chow‐fed controls at 3 months and was associated with an adverse metabolic phenotype. A, Body mass increased significantly more in fat‐fed mice compared with chow‐fed controls (45.1 ± 6.3 vs. 31.6 ± 0.9 g) *N* = 8 per group. B, Fasting blood glucose was significantly increased in fat‐fed mice compared with chow‐fed controls (6.4 ± 1.1 vs. 5.3 ± 0.03 mmol L^−1^) *N* = 6 per group. C, There was a trend of increased plasma insulin levels in fat‐fed mice compared with chow‐fed controls (3.1 ± 0.6 vs. 1.9 ± 0.3 ng mL^−1^) *N* = 6 per group. D, Homeostatic Model Assessment of Insulin Resistance (HOMA‐IR) was significantly increased in fat‐fed mice compared with chow‐fed controls (3.7 ± 1.1 vs. 1.3 ± 0.2) *N* = 6 per group. E and F, Glucose tolerance was significantly impaired in fat‐fed mice compared with chow‐fed controls – area under the curve (AUC) 73.7 ± 6.2 vs. 47.0 ± 2.4 *N* = 6 per group. G and H, Insulin sensitivity was significantly decreased in fat‐fed mice compared with chow‐fed controls – AUC 38.6 ± 4.7 vs. 18.7 ± 2.7 *N* = 6 per group. Data are presented as ±SEM (*P ≤ 0.05, **P ≤ 0.01, ***P ≤ 0.001).

Body mass gain was significantly higher in diet‐induced obese (DIO) mice compared with chow‐few controls (Figure 1A). By 3 months, DIO mice had developed an adverse metabolic phenotype characterized by increased fasting glucose concentration and higher Homeostatic Model Assessment of Insulin Resistance score (Figure 1B–D). DIO mice, in comparison with chow‐fed controls, were markedly glucose intolerant (Figure 1E,F) and insulin resistant (Figure 1G,H).

### Endothelial regeneration is preserved in mice with diet‐induced obesity

**Figure 2:**
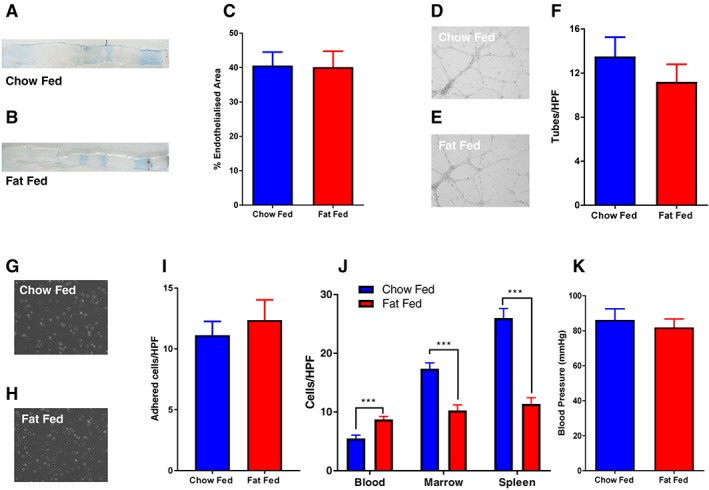
Endothelial regeneration remains preserved in mice with diet‐induced obesity at 3 months. A and B, Representative *in situ* Evans blue staining 7 d after vascular injury (blue staining indicates denuded endothelium). C, Endothelial regeneration 7 d after vascular injury was no different in fat‐fed mice when compared with chow‐fed controls (40.2 ± 4.6% vs. 40.6 ± 3.9%) *N* = 6 per group. D and E, Representative images of human umbilical vein endothelial cell (HUVEC) tube formation on matrigel after addition of conditioned media from MACs. F, There was no difference in tube formation of HUVEC exposed to conditioned media from myeloid angiogenic cells (MACs) from fat‐fed mice compared with chow‐fed controls (11.2 ± 1.6 vs. 13.5 ± 1.7) *N* = 10 fat and 15 chow. G and H, Representative images of adherent splenic‐derived MACs. I, There was no difference in adhesion of splenic‐derived MACs to fibronectin from fat‐fed mice compared with chow‐fed controls (12.3 ± 1.7 vs. 11.1 ± 1.1) *N* = 11 fat and 8 chow. J, Enumeration of MACs derived from blood, bone marrow and spleen by cell culture after 7 d. Blood‐derived MACs from fat‐fed mice were more abundant than those from chow‐fed controls (8.75 ± 0.49 vs. 5.5 0.5) *N* = 8. There were fewer marrow‐derived MACs from fat‐fed mice compared with chow‐fed controls (10.25 ± 0.95 vs. 17.4 ± 0.99) *N* = 8. Spleen‐derived MACs from fat‐fed mice were also less abundant than those from chow‐fed controls (11.38 ± 1.01 vs. 26 ± 1.7) *N* = 8. K, There was no difference in systolic blood pressure in fat‐fed mice compared with chow‐fed controls (82 ± 4.7 mmHg vs. 86.2 ± 6.2 mmHg) *N* = 11 per group. Data are presented as ±SEM (*P ≤ 0.05, **P ≤ 0.01, ***P ≤ 0.001). HPF = high‐power field.

Endothelial regeneration 7 d after endothelium‐denuding femoral artery wire injury did not differ significantly between DIO mice and chow‐fed controls after 3 months of feeding (Figure 2A–C). Pro‐angiogenic capacity of bone marrow‐derived MNCs from DIO mice was similar to that of chow‐fed mice (Figure 2D–F). Adhesion of angiogenic progenitor cells from DIO mice to fibronectin was preserved in comparison with that of cells from chow‐fed animals (Figure 2G–I). The abundance of MACs derived from blood was greater in DIO mice compared with chow‐fed controls (Figure 2J). However, splenic‐derived and bone marrow‐derived cells were fewer in DIO mice than in chow‐fed controls (Figure 2J). There was no difference in blood pressure between DIO mice and chow‐fed controls (Figure 2K).

To investigate whether prolonged exposure to obesity would affect endothelial regeneration, high‐fat feeding was continued for 6 months in a separate group of mice. After 6 months, a substantial difference in body mass persisted between DIO mice and chow‐fed controls (Figure S1A). However, fasting glucose and plasma insulin concentrations no longer differed between groups (Figure S1B‐C), perhaps reflecting age‐relating decline in glucose tolerance in chow‐fed mice. There was no significant difference in response to a glucose tolerance test (Figure S1D). Endothelial regeneration was not significantly different between DIO mice and chow‐fed controls after 6 months of feeding.

## Discussion

Patients undergoing percutaneous coronary intervention, particularly in obese and or insulin‐resistant settings, demonstrate reduced re‐endothelialization 5, 6, 7. Rapid re‐endothelialization is of critical importance to restore normal vascular function, reduce vascular inflammation and prevent adverse remodelling after percutaneous coronary intervention 2, 14. Successive reports of reduced abundance and functional impairment of circulating progenitor cells in obesity have led to the hypothesis that defective endothelial repair contributes to obesity‐related cardiovascular disease 10, 13, 15, 16, 17, 18, 19, 20, 21, 22. However, the current findings challenge these assumptions by demonstrating that endothelial regeneration remains preserved in mice with diet‐induced obesity.

By feeding a high‐fat diet for 3 months, we induced a markedly abnormal metabolic phenotype characteristic of glucose intolerance and insulin resistance in mice, characteristic of the metabolic profile of obese humans. We have previously shown that endothelial vasomotor function is altered by high‐fat feeding 23. Surprisingly, however, endothelial regeneration following mechanical arterial injury remained intact in obese mice despite the metabolic dysregulation. Following prolonged exposure to obesity for 6 months, endothelial regeneration remained unaltered between obese and chow‐fed mice. Differences in metabolic parameters had converged by 6 months, perhaps reflecting age‐related decline in glucose competence in chow‐fed mice. Although previous studies have demonstrated dysregulated vascular responses including impaired recovery from hind‐limb ischaemia in dietary obese mice 20, ours is the first study to investigate the effects of obesity on endothelial repair. It is noteworthy that genetic insulin resistance, in the absence of obesity, was associated with significant impairment of endothelial regeneration after experimental femoral artery injury in our previous study 5. The results of the current report indicate that the angiogenic response to femoral artery ligation and reparative response to femoral arterial denudation are differentially modulated by obesity. As yet, undefined compensatory mechanisms may offset the negative influence of insulin resistance on endothelial repair in dietary obese mice.

Of interest, an increased abundance of circulating MACs in obese mice was seen. These findings contrast with the reduced number of circulating endothelial progenitors that has been widely, although not universally, reported in prior clinical and experimental studies 8, 9, 10, 13. This comparison must be interpreted with caution, however, as most other studies quantified human or murine endothelial progenitors by flow cytometry as characterized by their expression of cell‐surface markers. Despite their association with cardiovascular disease, these circulating cells are not directly analogous to the MACs investigated in the current report, which are thought to contribute to endothelial regeneration through the release of locally acting pro‐angiogenic factors. No difference in the ability of conditioned medium from cultured MNCs to promote tubulogenesis *in vitro* was seen, suggesting that secretion of pro‐angiogenic cytokines is unaltered by obesity.

Although more numerous in the circulation in obese mice, MACs were less abundant in spleen‐derived or bone marrow‐derived MNC fractions, suggesting that endogenous stores are depleted in obesity – possibly through increased mobilization of cells into the circulation. Intriguingly, the opposite phenotype of reduced mobilization and circulating abundance of MACs was observed in genetically induced, rather than diet‐induced, insulin‐resistant mice 5. It is increasingly recognized that complex mechanisms, involving immune modulation in addition to insulin resistance, contrive to influence stem cell mobilization, differentiation and proliferation in obesity 24. However, the mechanisms by which obesity might regulate the abundance of circulating angiogenic progenitors have not yet been defined.

In conclusion, the findings of preserved endothelial regeneration despite metabolic dysregulation in dietary obese mice suggest that compensatory mechanisms mitigate the deleterious influence of insulin resistance on endothelial repair in obesity. The results of this study challenge the assumption that obesity has a universally negative influence on endothelial cell biology.

## Conflict of interest

The authors report no conflicts of interest.

## Author contributions

S. T. R., M. T. K., D. J. A. S. and S. B. W. conceived the study and contributed to the study design. N. J. H., S. T. R., A. A., N. Y. Y. and J. S. carried out experimental work and data collection and performed data analysis. N. J. H. and S. B. W. drafted the article, and all authors have reviewed it critically.

## Supporting information


**Supplementary Figure 1.** Metabolic status and endothelial regeneration in mice with diet‐induced obesity at six months. **A:** Body mass increased significantly in fat fed mice when compared to chow fed controls (44.55 ± 2.33 v 33.79 ± 1.23 g) *N* = 9 per group. **B:** Fasting blood glucose was no different between fat fed mice when compared to chow fed controls (7.55 ± 0.6 v 7.2 ± 0.76 mmol/L) *N* = 6 per group**. C:** There was no difference in fasting plasma insulin in fat fed mice when compared to chow fed controls (3.1 ± 0.6 v 1.9 ± 0.3 ng/ml) *N* = 6 per group. **D:** Glucose tolerance was no different in fat fed mice when compared to chow fed controls N = 6 per group. **E‐F:** Representative *in situ* Evans blue staining 7 days after vascular injury (blue staining indicates denuded endothelium). **G:** Endothelial regeneration 7 days after vascular injury was no different in fat fed mice when compared to chow fed controls (32.5 ± 3.26 v 30.5 ± 9.06) *N* = 5 per group. Data are presented as ^+^/_−_ S.E.M. (*P ≤ 0.05) (**P ≤ 0.01) (***P ≤ 0.001).
