# Supplementary figures and images for: Preservation of vascular endothelial repair in mice with diet‐induced obesity
Source: Obes Sci Pract. 2018 Jun 26;4(5):490–6. doi: 10.1002/osp4.282 (PMC6180714; doi:10.1002/osp4.282)

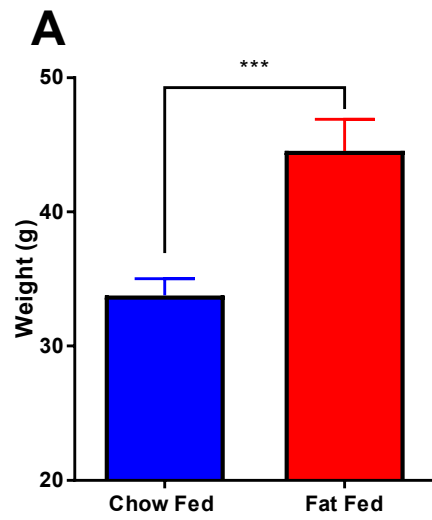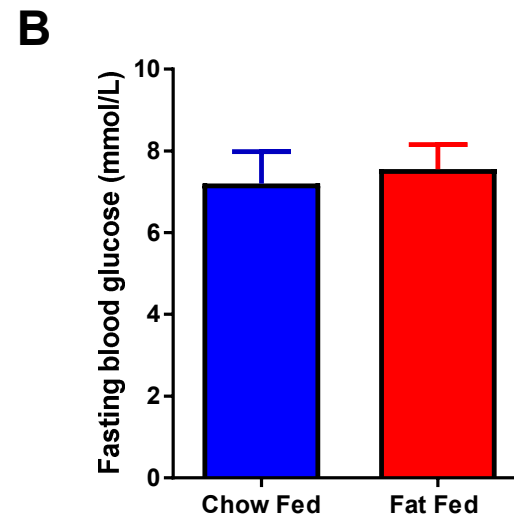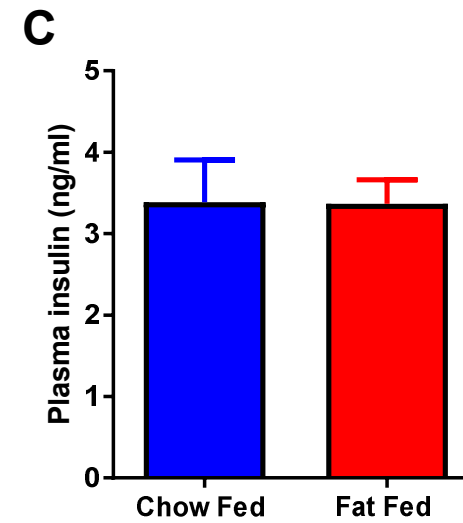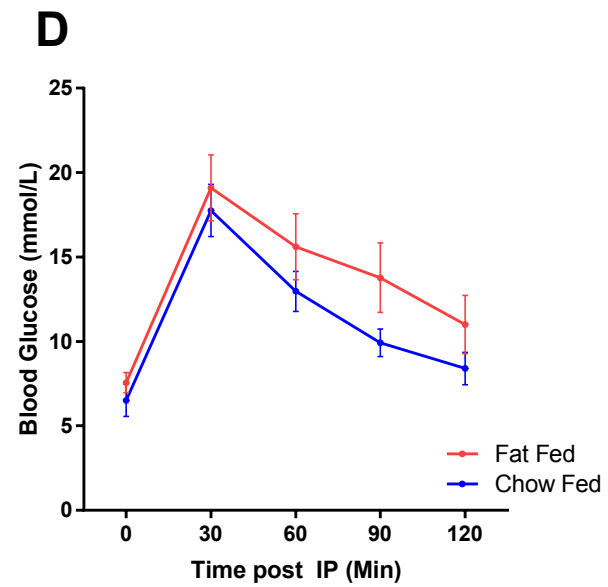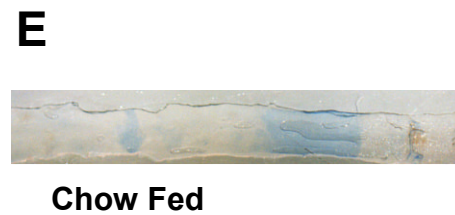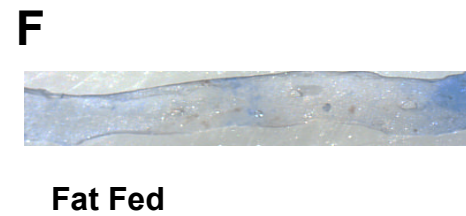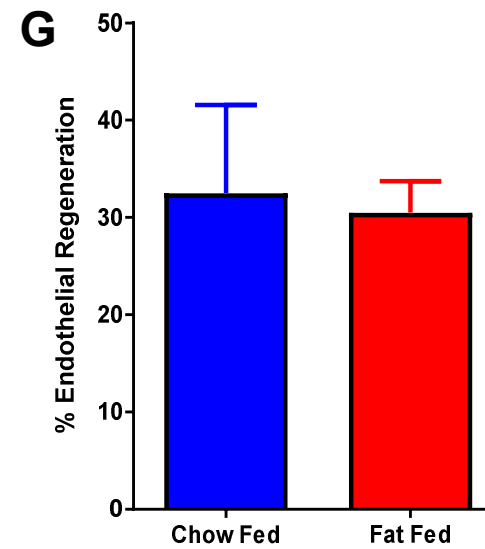

Supplement: Supplementary file 1 — Supplementary Figure 1. Metabolic status and endothelial regeneration in mice with diet‐induced obesity at six months. A: Body mass increased significantly in fat fed mice when compared to chow fed controls (44.55 ± 2.33 v 33.79 ± 1.23 g) N = 9 per group. B: Fasting blood glucose was no different between fat fed mice when compared to chow fed controls (7.55 ± 0.6 v 7.2 ± 0.76 mmol/L) N = 6 per group. C: There was no difference in fasting plasma insulin in fat fed mice when compared to chow fed controls (3.1 ± 0.6 v 1.9 ± 0.3 ng/ml) N = 6 per group. D: Glucose tolerance was no different in fat fed mice when compared to chow fed controls N = 6 per group. E‐F: Representative in situ Evans blue staining 7 days after vascular injury (blue staining indicates denuded endothelium). G: Endothelial regeneration 7 days after vascular injury was no different in fat fed mice when compared to chow fed controls (32.5 ± 3.26 v 30.5 ± 9.06) N = 5 per group. Data are presented as +/− S.E.M. (*P ≤ 0.05) (**P ≤ 0.01) (***P ≤ 0.001). [file OSP4-4-490-s001.pdf]
